# A New Sum Analogous to Gauss Sums and Its Fourth Power Mean

**DOI:** 10.1155/2014/139725

**Published:** 2014-05-21

**Authors:** Shaofeng Ru, Wenpeng Zhang

**Affiliations:** ^1^School of Economics & Management, Northwest University, Xi'an 710127, China; ^2^Department of Mathematics, Northwest University, Xi'an 710127, China

## Abstract

The main purpose of this paper is to use the analytic methods and the properties of Gauss sums to study the computational problem of one kind of new sum analogous to Gauss sums and give an interesting fourth power mean and a sharp upper bound estimate for it.

## 1. Introduction

Let *q* ≥ 3 be an integer, and let *χ* be a Dirichlet character mod⁡ *q*. Then for any integer *n*, famous Gauss sum *G*(*χ*, *n*) is defined as follows:

(1) $$\begin{array}{c} G\left(\chi ,n\right)=\overset{q}{\underset{a=1}{\sum }}\chi \left(a\right)\cdot e\left(\frac{na}{q}\right), \end{array}$$

where *e*(*y*) = *e* ^2*πiy*^.

This sum plays a very important role in the study of analytic number theory; many famous number theoretic problems are closely related to it. For example, the distribution of primes, Goldbach problem, the estimate of character sums, and the properties of Dirichlet *L*-functions are some good examples.

It is clear that if *χ* is a primitive Dirichlet character mod⁡ *q*, then we have $G(\chi ,n)=\bar{\chi }(n)\cdot G(\chi ,1)\equiv \bar{\chi }(n)\cdot \tau (\chi )$ and $\left|\tau \left(\chi \right)\right|=\sqrt{q}$. Many properties of *G*(*χ*, *n*) and *τ*(*χ*) can be found in [1–3].

In this paper, we introduce a new sum analogous to Gauss sums as follows:

(2) $$\begin{array}{c} G\left(\chi ,b,c,m;q\right)=\overset{q-1}{\underset{a=0}{\sum }}\chi \left(a^{2}+ba+c\right)\cdot e\left(\frac{ma}{q}\right), \end{array}$$

where *χ* is a character mod⁡ *q*, and *b*, *c*, and  *m* are any integers with (*m*, *q*) = 1.

The main purpose of this paper is to study the properties of *G*(*χ*, *b*, *c*, *m*; *q*), such as the following two problems:

(A) giving an upper bound estimate of *G*(*χ*, *b*, *c*, *m*; *q*);
(B) the problem of whether there exists a computational formula for the 2*k*th power mean
  (3) $$\begin{array}{c} \overset{q-1}{\underset{c=0}{\sum }}{\left|\overset{q-1}{\underset{a=0}{\sum }}\chi \left(a^{2}+ba+c\right)\cdot e\left(\frac{ma}{q}\right)\right|}^{2k},\;k\geq 2. \end{array}$$

It seems that no one has studied these problems yet; at least we have not seen any related results in the existing literature. The problems are interesting, because there exists a close relationship between the sum *G*(*χ*, *b*, *c*, *m*; *q*) and the generalized Kloosterman sums; they can also help us to further understand the properties of hybrid mean value of an exponential sum and character of a polynomial.

For general integer *q* > 3, these two problems seem to be hard to make progress. But if *q* = *p* > 2 is a prime and *k* = 2, then we can prove the following two conclusions.

**Theorem 1**

Let *p* be an odd prime and *χ* any nonprincipal character mod *p*. Then for any integers *b*, *c*, and *m* with (*m*, *p*) = 1, one has the estimate

(4) $$\begin{array}{c} \left|\overset{p-1}{\underset{a=0}{\sum }}\chi \left(a^{2}+ba+c\right)\cdot e\left(\frac{ma}{p}\right)\right|\leq 2\sqrt{p}. \end{array}$$

**Theorem 2**

Let *p* be an odd prime and *χ* any nonprincipal character mod *p*. Then for any integers *b* and *m* with (*m*, *p*) = 1, one has the identity

(5) ∑c=0p−1|∑a=0p−1χ(a2+ba+c)·e(map)|4={2p3−3p2−3p,if  χ  is  the  Legendre  symbol  mod p;2p3−6p2,if  χ  is  a  nonreal  character  mod p.

*Some Notes*. If *χ* = *χ*_0_ is the principal character mod *p*, then note that *χ*_0_(*a* ^2^ + *ba* + *c*) = 0 or 1 and |*G*(*χ*_0_, *b*, *c*, *m*; *p*)| ≤ 3. So, in this case, the result is trivial; we do not need to discuss problems (A) and (B).

For any integer *k* ≥ 3, whether there exists an exact computational formula for the 2*k*th power mean

(6) $$\begin{array}{c} \overset{p-1}{\underset{c=0}{\sum }}{\left|\overset{p-1}{\underset{a=0}{\sum }}\chi \left(a^{2}+ba+c\right)\cdot e\left(\frac{ma}{p}\right)\right|}^{2k}, \end{array}$$

is an open problem.

Furthermore, for general integer *q* > 3, whether there exists a nontrivial upper bound estimate for *G*(*χ*, *b*, *c*, *m*; *q*) is also an interesting problem.

## 2. Several Lemmas

In this section, we will give several lemmas, which are necessary in the proof of our theorems. Hereinafter, we will use many properties of character sums and Gauss sums; all of these can be found in [1, 4]. So they will not be repeated here. First we have the following.

**Lemma 3**

Let *p* be an odd prime; then, for any integer *c* with (*c*, *p*) = 1, one has the identity

(7) $$\begin{array}{c} \overset{p-1}{\underset{a=0}{\sum }}e\left(\frac{ca^{2}}{p}\right)=\left(\frac{c}{p}\right)\cdot \tau \left(\chi_{2}\right), \end{array}$$

where *χ*_2_ = (∗/*p*) denotes the Legendre symbol.

**Proof**

From the properties of Gauss sums and quadratic residue mod *p* we have

(8) ∑a=0p−1e(ca2p)=1+∑a=1p−1e(ca2p)=1+∑a=1p−1(1+(ap))·e(cap)=∑a=0p−1e(cap)+∑a=1p−1(ap)·e(cap)=(cp)∑a=1p−1(ap)·e(ap)=(cp)·τ(χ2).

This proves Lemma 3.

**Lemma 4**

Let *p* be an odd prime and *χ* any nonprincipal Dirichlet character mod *p*. Then for any integers *b* and *c*, one has the identity

(9) |∑a=0p−1χ(a2+ba+c)·e(ap)| =|∑r=1p−1χ¯χ2(r)·e((4c−b2)r−16¯·r¯p)|,

where $\bar{r}$ denotes the solution of the congruence equation *r* · *x* ≡ 1 mod *p*.

**Proof**

Since *χ* is a nonprincipal Dirichlet character mod *p*, from Lemma 3 and the properties of Gauss sums we have

(10) ∑a=0p−1χ(a2+ba+c)·e(ap) =1τ(χ¯)·∑a=0p−1∑r=1p−1χ¯(r)e(r(a2+ba+c)p)·e(ap) =1τ(χ¯)·∑r=1p−1χ¯(r)  ×∑a=0p−1e(ra2+(br+1)a+crp) =1τ(χ¯)·∑r=1p−1χ¯(r)  ×∑a=0p−1e(4¯r(2a+r¯(br+1))2+cr−4¯r¯(br+1)2p) =1τ(χ¯)·∑r=1p−1χ¯(r)  ×∑u=0p−1e(4¯ru2+4¯·(4c−b2)r−2¯·b−4¯·r¯p) =1τ(χ¯)·∑r=1p−1χ¯(r)(4¯·rp)·τ(χ2)  ·e(4¯·(4c−b2)r−2¯·b−4¯·r¯p) =τ(χ2)τ(χ¯)·χ¯(4)·e(−2¯·bp)·∑r=1p−1χ¯(r)(rp)  ·e((4c−b2)r−16¯·r¯p).

For any nonprincipal character *χ* mod *p*, we have $\left|\tau \left(\chi \right)\right|=\sqrt{p}$. So, from (10) and noting that $\left|\bar{\chi }\left(4\right)\right|=\left|e\left(-\bar{2}\cdot b/p\right)\right|=1$, we have

(11) |∑a=0p−1χ(a2+ba+c)·e(ap)| =|∑r=1p−1χ¯χ2(r)·e((4c−b2)r−16¯·r¯p)|.

This proves Lemma 4.

**Lemma 5**

Let *p* be an odd prime and *χ* any Dirichlet character mod *p*. Then for any integers *m* and *n*, one has the estimate

(12) $$\begin{array}{c} \overset{p-1}{\underset{a=1}{\sum }}\chi \left(a\right)\cdot e\left(\frac{ma+n\bar{a}}{p}\right)\leq 2\sqrt{p}\cdot {\left(m,n,p\right)}^{1/2}, \end{array}$$

where (*m*, *n*, *p*) denotes the greatest common divisor of *m*, *n*, and *p*.

**Proof**

This estimate is by Weil [5], Chowla [6], Malyshev [7], and Estermann [8] with some minor modifications.

**Lemma 6**

Let *p* be an odd prime; then, for any integer *n* with (*n*, *p*) = 1, one has the calculating formula

(13) ∑m=1p−1|∑a=1p−1χ(a)·e(ma+na¯p)|4={2p3−3p2   −3p−1,if  χ  is  the  principal  character  mod p;3p3−8p2,if  χ  is  the  Legendre's  symbol mod p;p2(2p−7),if  χ  is  a  nonreal  character  mod p.

**Proof**

See [9] or Corollary 2 of [10].

## 3. Proof of the Theorems

In this section, we will complete the proof of our theorems. First note that if *a* passes through a reduced residue system mod *p*, then *ma* also passes through a reduced residue system mod *p*, if (*m*, *p*) = 1. From these properties we have

(14) |∑a=0p−1χ(a2+ba+c)·e(map)| =|∑a=0p−1χ(m¯2a2+m¯ba+c)·e(ap)| =|χ2(m¯)∑a=0p−1χ(a2+mba+cm2)·e(ap)| =|∑a=0p−1χ(a2+mba+cm2)·e(ap)|.

So without loss of generality we can assume that *m* = 1. Now we prove Theorem 1. From Lemmas 4 and 5 we have

(15) |∑a=0p−1χ(a2+ba+c)·e(ap)| =|∑r=1p−1χ¯χ2(r)·e((4c−b2)r−16¯·r¯p)| ≤2p·(4c−b2,16¯,p)1/2=2p.

This proves Theorem 1.

Now we prove Theorem 2. From the properties of a complete residue system mod *p* we know that if *c* passes through a complete residue system mod *p*, then 4*c* − *b* ^2^ also passes through a complete residue system mod *p*. So from Lemma 4 we have

(16) ∑c=0p−1|∑a=0p−1χ(a2+ba+c)·e(ap)|4 =∑c=0p−1|∑r=1p−1χ¯χ2(r)·e((4c−b2)r−16¯·r¯p)|4 =∑m=0p−1|∑r=1p−1χ¯χ2(r)·e(mr−16¯·r¯p)|4 =∑m=0p−1|∑r=1p−1χ¯χ2(r)·e(mr+r¯p)|4.

If *χ* = *χ*_2_ is the Legendre symbol, then *χχ*_2_ = *χ*_0_ is the principal character mod *p*, so from (16) and Lemma 6 we have

(17) ∑c=0p−1|∑a=0p−1χ(a2+ba+c)·e(ap)|4 =∑m=0p−1|∑r=1p−1χ¯χ2(r)·e(mr+r¯p)|4 =1+∑m=1p−1|∑r=1p−1e(mr+r¯p)|4 =1+2p3−3p2−3p−1 =2p3−3p2−3p.

If *χ* is not a real character mod *p*, then from (16) and Lemma 6 we have

(18) ∑c=0p−1|∑a=0p−1χ(a2+ba+c)·e(ap)|4=∑m=0p−1|∑r=1p−1χ¯χ2(r)·e(mr+r¯p)|4=|∑r=1p−1χ¯χ2(r)·e(r¯p)|4 +∑m=1p−1|∑r=1p−1χ¯χ2(r)·e(mr+r¯p)|4=p2+p2(2p−7)=2p2(p−3).

Now combining (14), (17), and (18) we may immediately deduce the identity

(19) ∑c=0p−1|∑a=0p−1χ(a2+ba+c)·e(map)|4={2p3−3p2−3p,if  χ  is the Legendre symbol mod p;2p3−6p2,if  χ  is a nonreal character mod p.

This completes the proof of Theorem 2.
